# Early life Transepidermal Water Loss (TEWL) values as a predictor of food allergy and sensitisation at 2 years: results from the BASELINE Study

**DOI:** 10.1186/2045-7022-5-S3-O2

**Published:** 2015-03-30

**Authors:** Maeve Kelleher, Claire Culliane, Audrey Dunn Galvin, Deirdre Murray, Jonathan O B Hourihane

**Affiliations:** 1 Department of Pediatrics and Child Health, University College Cork, Cork, Ireland

## Rationale

Food Allergy is a growing public health concern. There is observational evidence that exposure to food allergens across a disrupted skin barrier can lead to food sensitisation (FS) and food allergy (FA). This mechanism of action has subsequently been proven in murine studies. We sought to ascertain whether a non invasive measurement of skin barrier function at 3 time points in early infancy could predict the development of food sensitisation and allergy in asymptomatic infants enrolled in an unselected prospective birth cohort.

## Methods

Infants on the BASELINE study attended for appointments in the early newborn period at 2, 6 and 12 months and 2 years. Transepidermal Water Loss (TEWL) was measured at birth, 2 and 6 months. Screening questions for food allergy were asked at each visit and investigated using modified EuroPrevall criteria for assessment of suspected food allergic reaction. At 2 years all infants had allergy screening by Skin Prick Test to a common panel of food allergens. Infants with positive SPT, unless previously safely tolerating the food allergen, underwent Oral Food Challenge (OFC) to differentiate food allergy from asymptomatic sensitisation.

## Results

1903 infants were enrolled onto the study from July 2009 to October 2012 with 1355 retained to 2 years. Mean Birth TEWL was 7.32 g water/m2/hr (±3.33) rising to a mean at 2 months of 10.97 gwater/m2/hr (±7.98) and then plateauing to a mean at 6 months of 10.71 gwater/m2/hr (±7.10). Sensitisation to any food at 2 years occurred in 6.27% (79/1260). Food allergy was confirmed in 4.45% (56/1258) of 2 year olds. There was no significant difference between birth TEWL values in infants with FS compared to those without or between those with FA or those without. The mean increased change between birth and 2 month TEWL values was significantly higher in infants with FA and FS and those without FA or FS. ["FS yes" Δ Birth - 2 months 6.36 (±9.48) g/_water_/m2 versus "FS no" 3.52 (±8.44) g/water/m2, p =0.007] and ["FA yes" Δ Birth - 2 months 6.43 (±10.47) g/_water_/m2 versus "FA no" 3.59 (±8.45) g/_water_/m2 p=0.02)].

## Conclusion

The prevalence of IgE mediated food allergy in Irish 2 year olds is 4.45%. Infants with FA at 2 years have a significantly increased change in TEWL from birth to 2 months compared to infants without FA. This suggests that maladaptation of the skin barrier from the inter-uterine environment through early infancy is associated with increased prevalence of FA. This signal for a development of food allergy in early life is seen in asymptomatic infants and may be amenable to intervention.

